# Anaplastic large cell lymphoma in children and adolescents

**DOI:** 10.1111/bjh.20154

**Published:** 2025-05-12

**Authors:** Eric J. Lowe, Wilhelm Woessmann

**Affiliations:** ^1^ Department of Pediatrics Eastern Virginia Medical School at Old Dominion University Norfolk Virginia USA; ^2^ Pediatric Hematology and Oncology and NHL‐BFM Study Center University Medical Center Hamburg‐Eppendorf Hamburg Germany

**Keywords:** anaplastic large‐cell lymphoma, anaplastic lymphoma kinase, children, diagnosis, therapy

## Abstract

Anaplastic lymphoma kinase (ALK)‐positive anaplastic large‐cell lymphoma (ALCL) accounts for >95% of ALCL cases in children and adolescents. The first description of ALCL as a CD30‐positive lymphoma in 1985 was followed by the detection of chromosomal translocations involving the *ALK* gene at chromosome 2p23. The pathogenesis of ALK‐positive ALCL is based on signalling from the constitutive active ALK kinase. The clinical characteristics, therapy regimens and outcome data were reported in the 1990s and 2000s. Different chemotherapy regimens led to astonishingly similar long‐term event‐free survival rates of 70%, independent of the drugs, doses and duration of therapy. Additionally, patients with relapsed ALCL are treated successfully with very different re‐induction and consolidation approaches leading to an overall survival approaching 95%. In the 2010s, minimal disseminated and residual disease, histological subtype and antibody titres against ALK were reported as significant independent prognostic factors. Over the last 15 years, targeted therapies (brentuximab vedotin, ALK inhibitors) have demonstrated high efficacy with low toxicity. The future of ALK‐positive ALCL is to define the role of targeted therapies by developing a ‘chemotherapy‐free’ approach for patients with standard‐risk ALCL and an integrated approach to cure patients with high‐risk ALCL.

## INTRODUCTION

Anaplastic large cell lymphoma (ALCL) accounts for 10%–15% of childhood and adolescent non‐Hodgkin lymphomas (NHL).^1^, ^2^ The WHO classification distinguishes ALK‐positive ALCL, ALK‐negative ALCL, breast implant‐associated ALCL and cutaneous CD30‐positive lymphoproliferations (cutaneous ALCL and lymphomatoid papulosis).^3^ Greater than 95% of ALCLs in children and adolescents are ALK‐positive ALCLs.^4^, ^5^ Therefore, this review focuses on ALK‐positive ALCL, emphasizing the compilation of insights derived from pathological characteristics, clinical features and therapeutic approaches, with the aim of delineating current clinical needs and future research directions.

## HISTORY AND PATHOBIOLOGY OF ALK‐POSITIVE ALCL

ALCL was first described by Stein et al. in 1985 as a CD30‐positive lymphoma subtype with anaplastic so‐called ‘hallmark’ cells.^6^ During the following years, several groups reported the association of translocation t(2;5)(p23;q35) with ALCL.^7^, ^8^, ^9^ In 1994, Morris et al. cloned the involved genes, demonstrating that the *nucleophosmin‐1 (NPM1)* gene on chromosome 5 was fused to the *anaplastic lymphoma kinase (ALK)* gene on chromosome 2.^10^ The ubiquitously active *NPM1*‐promoter drives the expression of the resulting *NPM1::ALK* fusion gene. In time, the oncogenic mechanisms of the fusion gene *NPM1::ALK* have been deciphered.^11^, ^12^, ^13^ The dimerization of two NPM1::ALK proteins mediated by the N‐terminal NPM1 domain leads to autophosphorylation and constitutive activation of the C‐terminal ALK kinase domain. This resulting activation of signalling pathways, including the JAK‐STAT3, Raf‐MEK‐ERK, PI3K‐AKT, STAT5 and PLC gamma pathways, is heavily involved in cell survival, inhibition of apoptosis, cell cycle progression and immune evasion. While the *NPM1::ALK* fusion accounts for 90% of ALK‐positive ALCL in children and adolescents,^4^, ^5^, ^14^ more than 20 variant (i.e. non‐*NPM1*) *ALK* fusion partners have been reported, with *TPM3* and *ATIC* accounting for two‐thirds of the variant partner genes.^15^ For further information on the pathobiology of ALK‐positive ALCL, please refer to excellent reviews focusing on this issue.^11^, ^12^, ^13^, ^16^

A critical historical milestone in diagnosing ALCL was the development of the ALK‐1 monoclonal antibody by Karen Pulford.^17^ Since ALK is predominantly expressed in a limited number of scattered neurons and is not detected in haematopoietic tissue postnatally,^18^ the presence of ALK expression in lymphoid cells serves as a diagnostic marker for ALK‐positive lymphoma. The establishment of a reproducible pathological definition facilitated significant advancements by ensuring consistency in identifying, treating and researching ALK‐positive ALCL. In addition, this defining characteristic ultimately led to the recognition of ALK‐positive ALCL as a distinct lymphoma in the WHO Classification of Tumours beginning in 2008.^3^

## CLINICAL CHARACTERISTICS

**FIGURE 1:**
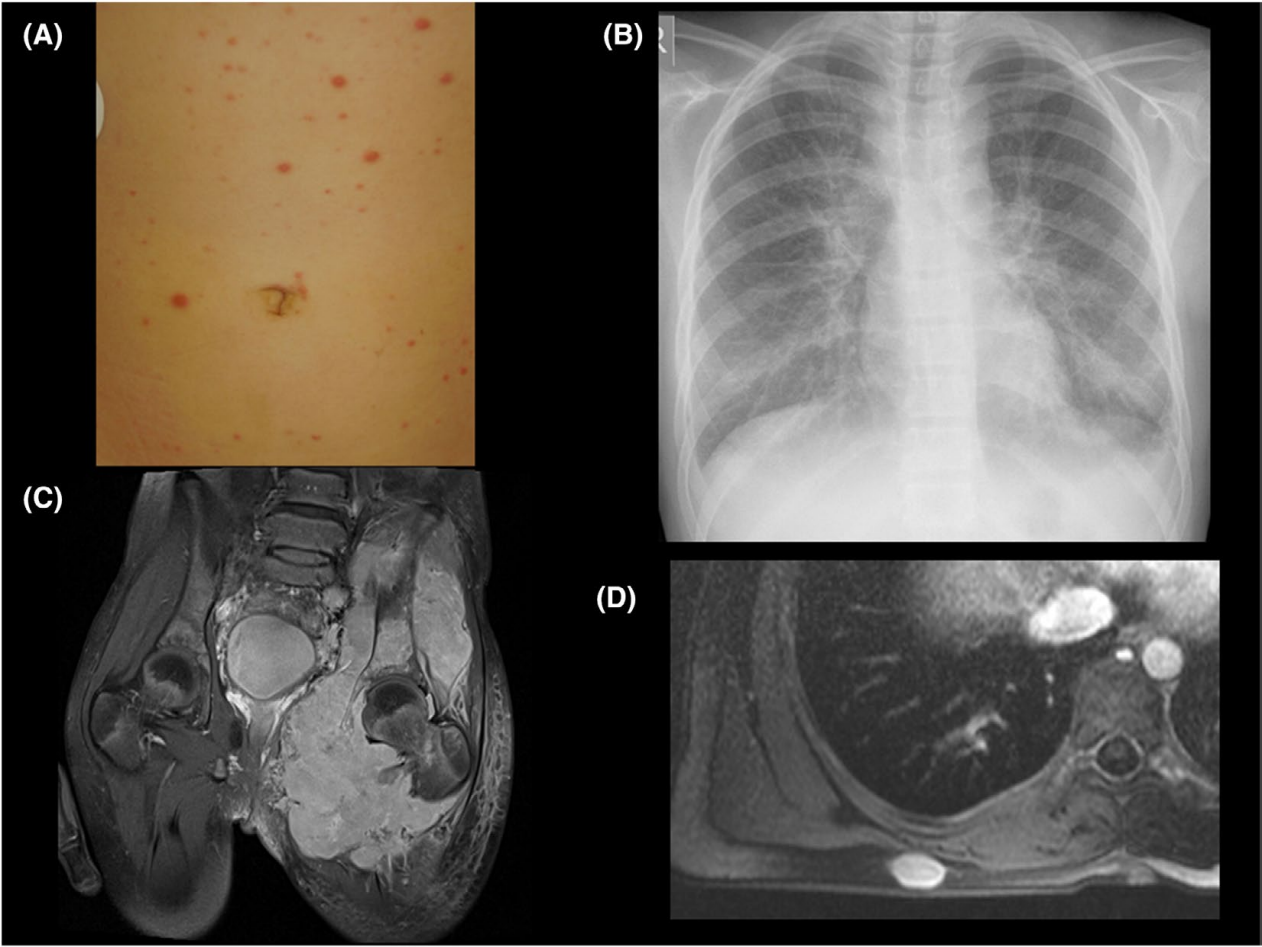
Clinical presentations of children with anaplastic lymphoma kinase‐positive anaplastic large‐cell lymphoma. (A) Skin involvement, (B) lung involvement, (C) bone and soft tissue, (D) subcutaneous soft tissue lesion.

ALK‐positive ALCL occurs most commonly in the first four decades of life with a slight male predominance. The median age at diagnosis for children and adolescents with ALCL is approximately 12 years of age (range: 0.2–18 years) with rare cases in the first year of life. Children with ALK‐positive ALCL rarely present with stage I disease with most patients having advanced staged disease at diagnosis (II, 20%; III, 60%–65%; IV, 10%).^2^, ^19^, ^20^, ^21^, ^22^, ^23^, ^24^, ^25^ The frequency of bone marrow involvement varies between 5% and 15% depending on the method of detection.^2^, ^19^, ^20^, ^21^, ^22^, ^23^, ^24^, ^25^ The central nervous system (CNS) is rarely involved (1%–3%), but among primary CNS lymphomas in children, ALCL accounts for 25%–30% of cases.^26^, ^27^ Although lymph nodes are affected in 85%–90% of children, 65%–75% of children have extranodal disease (Table 1) and approximately 70% present with B symptoms.^2^, ^19^, ^20^, ^21^, ^22^, ^23^, ^24^, ^25^ While the International Pediatric NHL Staging System (IPNHLSS) represents an improvement over the traditional St. Jude staging criteria, it continues to be challenging to accurately stage a disease characterized by the frequent presence of extranodal involvement. In addition to making staging difficult, extranodal disease and B symptoms explain how ALCLs mimic infectious, inflammatory diseases or other malignancies. For example, ALCL may mimic varicella (Figure 1A), interstitial pneumonia (Figure 1B) or even Ewing sarcoma (Figure 1C). Subcutaneous lesions may appear as soft tissue sarcomas (Figure 1D).

**TABLE 1:** Sites of disease involvement in children with ALK‐positive anaplastic large‐cell lymphoma.

Site of disease	Frequency (%)
Lymph nodes	85–95
Mediastinum	25–45
Skin	15–25
Bone	15–20
Lung	15–20
Liver	20–30
Spleen	15–20
Soft tissue	10–20
Bone marrow	5–15
Central nervous system	1–3

Abbreviation: ALK, anaplastic lymphoma kinase.

A few unique clinical manifestations of ALK‐positive ALCL are worth mentioning:

- ALCL presenting with concomitant haemophagocytic lymphohistiocytosis (HLH). ALCL is very immunoreactive, and cytokines like the soluble interleukin (IL)2 receptor or IL6 often are highly elevated in the serum of patients^28^ and may be associated with haemophagocytosis/macrophage activating syndrome.^29^ In cases of EBV‐negative haemophagocytosis in children above 1–2 years of age, an ALCL should be excluded, even in patients without lymphadenopathy.
- ALCL may present with a ‘waxing and waning’ pattern in some children, although the exact frequency of this presentation is unknown.^22^, ^30^ Affected children may initially present with enlarged lymph nodes or a soft tissue tumour consistent with lymphoma, which spontaneously regresses but later recurs after several weeks or, in rare instances, even months.
- A leukaemic presentation, defined by the presence of cytologically circulating lymphoma cells, is rare.^31^ It is associated with the small cell variant.^32^

## PATHOLOGICAL CHARACTERISTICS

By definition, ALCL is CD30 positive. ALK‐positive ALCL expresses ALK‐fusion proteins. Although at least one T‐cell marker is detectable in almost all ALCL, the lymphomas typically show a reduced T‐cell marker profile, which led to the historical nomenclature of a so‐called ‘null cell phenotype’.^3^ A clonally rearranged T‐cell receptor can be demonstrated. In addition, cytotoxic molecules TIA1, Granzyme B and perforin are expressed in most cases. Histologically, the common subtype with abundant hallmark cells is prevalent in about 65% of childhood and adolescent cases, the small cell variant accounts for 6% and the lymphohistiocytic variant for 2%–3% of cases. 25% of ALCL show a mixed pattern with small cell or lymphohistiocytic components.^4^, ^16^

## IMMUNE RESPONSE AGAINST ALK‐POSITIVE ALCL

ALCL is a highly immunogenic lymphoma characterized by elevated levels of pro‐inflammatory cytokines at diagnosis.^28^ Patients with ALK‐positive ALCL produce humoral and cellular immune responses against the oncoantigen ALK.^33^ Both ALK‐specific antibodies and ALK‐specific CD8 and CD4 T‐cells can be detected in patients.^34^, ^35^, ^36^, ^37^, ^38^, ^39^ This immunoactivity is reflected in a high proportion of patients presenting with B symptoms or even haemophagocytosis.^2^, ^19^, ^22^, ^29^, ^30^ Some patients even seem to temporarily control the lymphoma as evidenced by a ‘waxing‐and waning’ clinical course.^22^, ^30^ Despite this immunogenicity, the universal expression of programmed death‐ligand 1 (PD‐L1) likely induces an immunosuppressive microenvironment.^13^, ^40^, ^41^

The immune response may play a role in the final control of ALCL as the strength of the immune response measured by the ALK antibody titre inversely correlates with the risk of relapse.^36^, ^42^, ^43^ Furthermore, immunization with ALK peptides or cDNA has been effective prophylactically and therapeutically in mouse models of ALK‐rearranged tumours.^44^, ^45^ In addition, the low further relapse rate of relapsed ALCL after allogeneic HSCT of only 10–20% hints towards a graft‐versus‐ALCL effect.^46^, ^47^, ^48^, ^49^, ^50^, ^51^

## LESSONS FROM CHEMOTHERAPY STUDIES BEFORE TARGETED THERAPY

Following the identification of ALCL as a specific lymphoma, prospective trials with different chemotherapy backbones were performed.^2^, ^19^, ^22^, ^23^, ^24^, ^25^, ^52^ Several groups utilized different short‐pulse multiagent chemotherapy courses over a period of 5–6 months initially developed for mature B‐cell lymphomas. In contrast, others used acute lymphoblastic leukaemia (ALL)‐type therapy or intensive T‐cell lymphoma/leukaemia regimens. In North America, a specific APO regimen (doxorubicine, vinvristine, prednisone, 6‐merccaptopurine and low‐dose methotrexate) was developed with a treatment duration of 1 year. Despite different regimens, drugs, doses and treatment durations, the event‐free (EFS) or progression‐free survival (PFS) rates were astonishingly similar, between 65% and 75% (Table 2).

**TABLE 2:** Event‐free or progression‐free survival of trials in children and adolescents as well as adults with ALK‐positive anaplastic large‐cell lymphoma.

Trial	Age	Number of patients	Regimen	EFS or PFS at 2–5 years (%)	Reference
HM90/91	C, Adol	82	B‐NHL (LMB) with COPADM + maint.	66 ± 10	19
NHL‐BFM 90	C, Adol	89	B‐NHL (BFM)	76 ± 5	24
UKCCSG	C, Adol	72	B‐NHL (LMB)	59 ± 12	25
EICNHL:ALCL99	C, Adol	487	B‐NHL (BFM) (methotrexate and vinblastine randomization)	73 ± 4	20, 53, 54
POG9315	C, Adol	86	APO	72 ± 6	52
CCG‐5941	C, Adol	86	T cell (compressed to 1 year)	68 ± 11	21
COG: ANHL0131	C, Adol	125	APO (vinblastine randomization)	76 ± 7	55
AIEOP: LNH‐92	C, Adol	34	T‐ALL	65 ± 8	23
AIEOP: LNH‐97	C, Adol	32	B‐NHL (intensified)	68 ± 8	56
DSHNHL: CVHO(E)P	Adults	78	CHO(E)P	76 ± 10	57
GELA: LNH87, 93, 98	Adults	64		72 (58–83)	58
Combination of chemotherapy with BV					
COG: ANHL12P1	<22 years	68	ALCL99 + BV	79 (67–87)	59
ECHELON‐2 (peripheral T cell lymphomas)	Adults	98	Randomization CHOP versus A+CHP	A+CHP: HR 0.4	60, 61
Combination of chemotherapy with Crizotinib					
COG: ANHL12P1	<22 years	66	ALCL99 + Crizotinib	77 (69–88)	62

Abbreviations: Adol, adolescents; ALK, anaplastic lymphoma kinase; BV, brentuximab vedotin; C, children; EFS, event‐free survival; HR, hazard ratio; PFS, progression‐free survival.

In the 2000s, randomized trials enrolling a higher number of patients based on the Berlin–Frankfurt–Muenster (BFM) B‐cell backbone (EICNHL: ALCL99) and the APO backbone were performed.^20^, ^21^, ^53^, ^55^ To date, the international ALCL99 trial is the largest clinical trial for paediatric ALCL. It replicated the EFS of 74% of the NHL‐BFM 90 trial in a larger number of patients. The study also demonstrated that intrathecal chemotherapy is dispensable if intravenous methotrexate at 3 g/m^2^ as a 3‐hour infusion is used instead of 1 g/m^2^ given over 24 hours.^20^ Moreover, adding vinblastine did not improve the EFS in clinically defined high‐risk patients, although the longer duration of therapy postponed relapses.^53^, ^63^ Interestingly, adding vinblastine to the APO regimen did not result in a lower relapse rate in the COG study, confirming the results of ALCL99.^55^ Furthermore, the studies NHL‐BFM 95 and LNH‐97 applied an intensified BFM regimen with high‐dose methotrexate (5 g/m^2^) and high‐dose cytarabine and etoposide to children clinically defined as ‘high risk’ without improvement of EFS.^56^, ^64^ Children with completely resected stage I disease were cured with only three courses of chemotherapy in the ALCL99 trial.^65^ Local therapy may even suffice if ALK‐positive ALCL is limited to a single resected skin lesion without detection of minimal disseminated disease.^66^

These data cumulatively demonstrate that intensification of chemotherapy does not increase EFS and indicate that no single chemotherapy regimen has demonstrated superiority. Across all regimens, EFS rates plateau at approximately 70%.^54^ The findings suggest that the development of traditional chemotherapy for ALCL has likely reached its therapeutic limits, underscoring the need for alternative or targeted treatment strategies. In addition, prognostic factors are necessary to separate standard risk patients amenable to therapy reduction from high‐risk patients needing additional experimental treatments.

## LESSONS FROM RELAPSE THERAPY BEFORE TARGETED THERAPY

Treatment for recurrent or refractory disease varied widely from single‐agent chemotherapy over autologous haematopoietic stem‐cell transplantation (HSCT) to allogeneic HSCT, with different strategies having some success.^67^, ^68^, ^69^ Conditioning regimens for allogeneic HSCT often were total body irradiation (TBI)‐based.^67^, ^68^, ^69^ High‐dose therapies before autologous HSCT were heterogeneous and included TBI‐, Busulfan‐based ones as well as BCNU, etoposide, cytosinarabinoside and melphalan (BEAM).^67^, ^68^, ^69^

Time to relapse from initial diagnosis, CNS disease and bone marrow involvement at relapse were identified as the most significant prognostic factors.^67^, ^69^ In addition, patients with relapse of a CD3‐positive ALCL had a high risk of failure after autologous HSCT.^69^ Based on this information, the EICNHL performed a prospective study testing the efficacy of consolidation with an allogeneic HSCT after conditioning with TBI, thiotepa and etoposide for high‐risk relapse (those with progression during therapy or CD3‐positive ALCL), consolidation with an autologous HSCT after high‐dose therapy with BEAM for medium‐risk relapse (relapse of a CD3‐negative ALCL after initial treatment but before 1 year from diagnosis) and vinblastine monotherapy for 2 years in patients with a late relapse (>12 months from initial diagnosis). The study confirmed the high efficacy of an allogeneic HSCT after TBI‐based conditioning for high‐risk relapsed ALCL with an EFS of 80% and vinblastine monotherapy for low‐risk relapsed ALCL with an EFS >80%. However, the EFS of less than 40% after high‐dose therapy with BEAM and an autologous HSCT was far below expectations and is no longer recommended as consolidation for children with relapsed ALCL.^49^ In summary, the low relapse rate after allogeneic HSCT compared to autologous HSCT suggests a strong graft‐versus‐ALCL effect, and the effectiveness of vinblastine monotherapy for late relapses after intensive chemotherapy suggests that less toxic chemotherapy could be utilized for patients with low‐risk disease.

In light of the development of new targeted drugs discussed below, the indication for consolidation of children with relapsed ALCL with an allogeneic HSCT, with its risk of mortality as well as the associated risks of GvHD and late effects, is to be questioned. However, allogeneic HSCT is currently the only consolidation with a proven high chance of cure for children with refractory or early relapsed ALCL. Regarding a possible reduction of late effects of an allogeneic HSCT, data on reduced toxicity conditioning regimens with comparable efficacy to myeloablation for a consolidative allogeneic HSCT are emerging.^46^, ^70^, ^71^

## RECENT THERAPEUTIC OPTIONS

**FIGURE 2:**
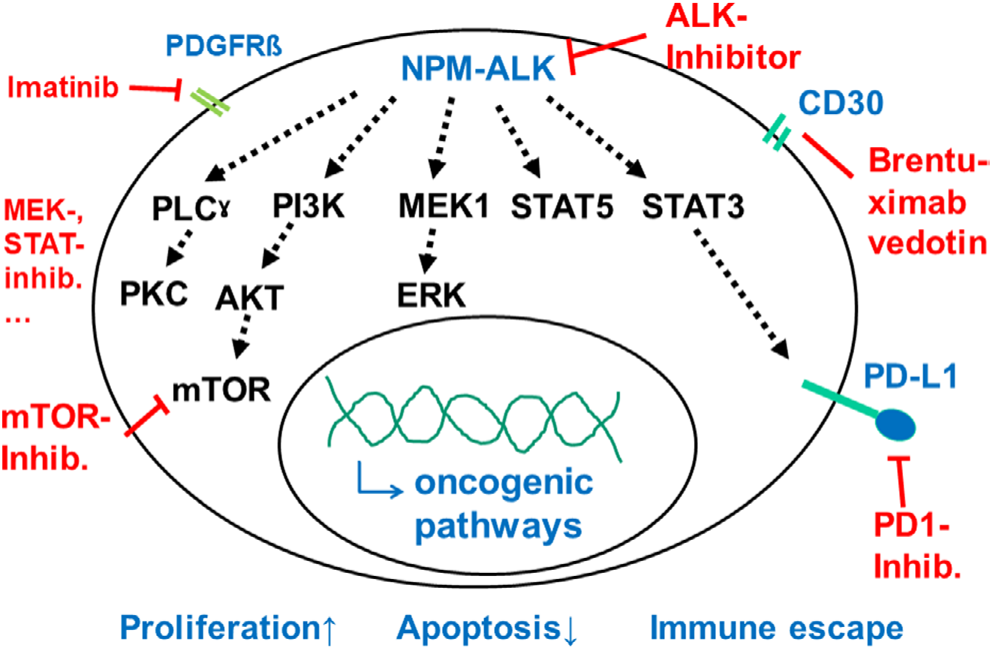
Signalling from the constitutively activated ALK in NPM‐ALK‐positive ALCL with possible therapeutic targets. ALCL, anaplastic large cell lymphoma; ALK, anaplastic lymphoma kinase; NPM, nucleophosmin.

The uniform expression of CD30 in ALCL and the pathogenic role of the activated ALK and its signalling offer therapeutic targets that specifically target ALK‐positive ALCL (Figure 2, Table 3).

**TABLE 3:** Response, further therapy and outcome of patients with refractory or relapsed ALK‐positive ALCL to ALK‐inhibitor monotherapy in early clinical trials, compassionate use programmes or retrospective analyses.

ALK inhibitor	Study type	Age (years)	Number of patients (*N*)	ORR (CR + PR) (%)	Number of patients who received allogeneic HSCT for consolidation	Stop in CR without further therapy, *N*, Outcome (FUP)	PFS (2–3 years) (%)	Reference
Crizotinib	Phase I/II	3–20	26	86	13	0	n.a.	72, 73
	Comp	15–37	9	100	3	0	n.a.	74
	Phase II	18–83	12	83	1	0	65	75
	Retro	15–82	25	82	1	0	57	76
	Phase II	1–60	25	67	7	2 2 CR (FUP 7 + 9 m)	40	77
Alectinib	Phase II	6–70	10	80	3	0	58	78
Ceritinib	Phase I	Children	8	75	5	0	67	79
Brigatinib	Comp	19–73	15	93	7	1 1CR (FUP 0.4y)	73	80
Crizotinib Ceritinib Lorlatinib Alectinib	Retro	<22	At first relapse: Crizotinib (34) Ceritinib (1) Lorlatinib (2) At any relapse: Crizotinib (48) Ceritinib (1) Lorlatinib (7) Alectinib (3)	95	21	10 9 CR, 1 Relapse (FUP 0.3–7.1 years)	n.a.	81
Crizotinib Ceritinib Lorlatinib	Retro	<18	At first relapse: Crizotinib (4) Ceritinib (2) At any relapse: Crizotinib (4) Lorlatinib (1)	73	n.a.	n.a.	n.a. (all 8 responders in CR)	82

Abbreviations: ALCL, anaplastic large cell lymphoma; ALK, anaplastic lymphoma kinase; comp, compassionate use; CR, complete response; FUP, follow‐up period; PFS, progression‐free survival; retro, retrospective.

### Brentuximab Vedotin (BV)

Brentuximab vedotin is generated by modifying the anti‐CD30 monoclonal antibody, SGN‐30, by adding a linker which then attaches to monomethyl auristatin E, a synthetic anti‐neoplastic agent that binds tubulin. The specificity to CD30 and the fact that tubulin inhibitors are known to be very active in ALCL make BV an attractive agent to treat ALCL. In a phase II study utilizing BV in adults with relapsed or refractory (R/R) ALCL, 86% of patients had an objective response, and 66% achieved a complete response with ALK‐positive and ALK‐negative disease having similar response rates.^83^, ^84^ The toxicity of BV was manageable, and although 33/58 (57%) of patients experienced peripheral neuropathy, 91% of these patients had resolution or improvement of their neuropathy. This study led to the FDA approval of BV for adults with relapsed or refractory ALCL in 2011.

Following the demonstration of efficacy in adult patients, a phase 1/2 study evaluating BV in paediatric patients with R/R ALCL was performed. Of the 17 patients enrolled, 53% achieved a response (41% complete remission (CR), 12% partial response (PR)) and 13 ultimately received HSCT.^85^ Only 8% of patients had a drug‐related serious adverse event, and while 33% of patients experienced peripheral neuropathy, only one had severity ≥grade 3. This trial demonstrated tumour response to BV monotherapy with a manageable safety profile in children/adolescents with ALCLs.

The high response rate as a single drug with tolerable toxicity in both adults and children led to testing BV in combination with chemotherapy in newly diagnosed patients.^59^, ^60^, ^61^ The ECHELON‐2 study tested the efficacy of brentuximab vedotin with CHP (A+CHP, BV + cyclophosphamide, doxorubicine and prednisone) versus the standard CHOP (Vincristine + CHP) regimen in newly diagnosed adult patients with CD30‐positive NHL. Of 452 patients, 316 (70%) had systemic ALCL (ALK‐positive, *n* = 98). A+CHP led to a statistically higher OS and PFS compared to CHOP (HR for OS = 0.66, *p* = 0.02; HR for PFS = 0.71, *p* = 0.01). For patients with systemic ALCL, the risk of a PFS event was reduced by 41% for those receiving A+CHP (*p* = 0.01). This international trial set A+CHP as the new standard of care for adults with systemic ALCL.^60^, ^61^

The BV arm of a randomized phase II clinical trial examined the tolerability, EFS and OS of adding BV to ALCL99 chemotherapy in children and adolescents with systemic ALK‐positive ALCL.^59^ Patients received BV 1.8 mg/kg on day 1 of each 21‐day cycle for six cycles. In 68 patients with a median age of 12 years (range: 2–21 years), the 2‐year EFS was 79%, and the OS was 97%, with no patient relapsing while receiving therapy. Like prior paediatric studies in ALCL, the primary grade 3–4 toxicities included haematological events, mucositis, and febrile neutropenia. Still, there were no toxic deaths and no cases of grade 3 or 4 peripheral neuropathy. The addition of BV did not seem to increase toxicity, and the EFS and OS compare favourably with all previous clinical trials, resulting in this regimen as one of the standards of care for paediatric ALCL.^59^

### ALK inhibitors

ALK‐positive ALCL is dependent on signalling from a constitutively activated ALK. After the tolerability and efficacy of ALK inhibitors were demonstrated in adults with ALK‐rearranged non‐small cell lung cancer, these drugs were consecutively tested in clinical trials or compassionate use programmes in children, adolescents and adults with ALCL^46^, ^72^, ^73^, ^74^, ^75^, ^76^, ^79^, ^80^ (Table 3). The first‐phase 1/2 paediatric trial enrolled 26 paediatric patients with R/R ALK‐positive ALCL. In this study, patients responded to both doses of crizotinib, 165 mg/m^2^/dose twice daily and 280 mg/m^2^/dose twice daily, with an objective response rate of 83% with the lower dose and 90% with the higher dose, indicating sensitivity to crizotinib.^72^, ^73^ Twenty‐three of 26 patients had a complete or partial response, and 18 had their response within 4 weeks of initiating treatment. Based on the results and a low toxicity profile, crizotinib was approved in the United States in 2021 and in the EU in 2022 for children and adolescents with R/R ALK‐positive ALCL.

The French phase 2 ACSÉ trial tested for the first time the efficacy of a limited duration of Crizotinib therapy for children and adults with relapsed ALK‐positive ALCL.^77^ The response rate of the 24 evaluable patients at 8 weeks was 67%. All patients discontinued the drug after 0.4–47 months due to either toxicity, a planned allogeneic HSCT, by choice or for a relapse. The 3‐year PFS was 40% with only one patient off therapy for 6 months without any further therapy and without relapse. These data suggest that a limited time of treatment with Crizotinib does not lead to a cure in most patients.

Given the efficacy and tolerability of crizotinib in relapsed ALCL, the phase 2 ANHL12P1 trial examined the efficacy and safety of crizotinib in combination with chemotherapy in paediatric patients with newly diagnosed ALK‐positive ALCL.^62^ The study enrolled 66 patients with a median age of 14 years. The results demonstrated a 2‐year EFS rate of 77% and an OS rate of 95%, comparable to the best outcomes reported to date. However, 20% of patients in the crizotinib arm experienced a grade 2 or higher thromboembolic event. This significant adverse event rate complicates any recommendation for this particular crizotinib combination, despite the demonstrated therapeutic activity.

Crizotinib has also been combined with vinblastine in the CRISP trial and on an individual basis in 13 children with R/R ALCL. Despite responses in all patients, severe grade 3–4 mainly haematological and infectious but also gastrointestinal toxicities were observed so that the trial was closed and the combination can no longer be recommended.^86^

Additional next‐generation ALK inhibitors, developed following crizotinib's success, have been used in children and adolescents with relapsed ALCL. In an open‐label phase 2 trial with paediatric and adult patients, an objective response to alectinib was observed in eight of 10 patients (80%), with six complete responses.^46^ The 1‐year PFS and OS rates were 58% and 70% respectively. Ceritinib had a response in six of eight paediatric patients with R/R ALCL.^79^ Both drugs showed acceptable safety profiles.^46^, ^79^ An abstract on part 1 of the BrigaPED study reports responses in all nine patients with R/R ALCL with limited toxicity and definition of a recommended phase 2 dose.^87^ Therefore, in paediatric patients with ALK‐positive ALCL, crizotinib, alectinib and ceritinib have demonstrated efficacy in clinical trials.

Real‐world data on ALK inhibitors are emerging from retrospective analyses. Marks et al. reported on 81 patients with relapsed ALK‐positive ALCL.^81^ Crizotinib was the most common ALK inhibitor used at first relapse (*n* = 34) with an excellent CR rate of 92%. In addition, all three patients who received crizotinib during initial therapy and were rechallenged with the drug for relapsed disease achieved a sustained CR. Including subsequent relapses and therapy, at some point, 48 patients received crizotinib, 7 lorlatinib, 3 alectinib and 1 ceritinib. Importantly, of the 10 patients who discontinued ALK inhibitor therapy while in remission, nine remain in remission. This contrasts with other reports of two patients progressing within 20 days of stopping crizotinib.^88^

Future therapeutic strategies for patients with ALCL should consider the differences between ALK inhibitors. For example, crizotinib exhibits limited CNS penetration whereas second‐ and third‐generation inhibitors show significant penetration into the CNS.^89^ Moreover, the newer generation of ALK inhibitors may overcome resistance associated with gatekeeper mutations.^90^, ^91^ However, these insights should be interpreted with caution, as the majority of supporting data are from studies in ALK‐positive non‐small cell lung cancer rather than ALCL.

### Checkpoint inhibitors

Immune checkpoint inhibition represents another promising treatment modality for ALK‐positive ALCL given the PD‐L1 expression and the role of the host immune response in disease control. Case reports have demonstrated favourable responses to the PD‐1 inhibitors nivolumab or pembrolizumab in patients with R/R ALK‐positive ALCL.^92^, ^93^ Additional research to elucidate the efficacy of checkpoint inhibitors is currently being investigated in the NIVO‐ALCL trial (NCT03703050) utilizing nivolumab monotherapy in patients with R/R ALCL.

#### Other new approaches

Activated ALK induces several signalling pathways that could be targeted with available drugs (Figure 2).^11^, ^12^, ^13^, ^16^, ^94^ However, BV, ALK inhibitors and checkpoint inhibitors are highly effective and have not even been explored in detail in this rare disease, so that clinical development of other targets during the next decade seems unlikely.

Vaccination with ALK epitopes is an effective consolidation in mouse models of both ALCL and lung cancer (see above).^44^, ^45^, ^95^ However, clinical development to boost the existing anti‐ALK immune response in patients has not started yet.

## PROGNOSTIC FACTORS

Analyses from the 1990 described clinical risk factors (involvement of lung, liver, spleen, mediastinum, skin)^63^ which were used for risk stratification in the ALCL99 trial.^53^ Minimal disseminated disease (MDD), minimal residual disease (MRD), low anti‐ALK‐antibody titres, and small‐cell or lymphohistiocytic components (histological subtype) had since been shown as independent poor prognostic factors in the 2000s and later been validated (Table 4).^4^, ^36^, ^42^, ^43^, ^59^, ^96^, ^97^, ^98^, ^99^, ^100^ Minimal disease is determined in reference laboratories by quality‐controlled qualitative reverse transcriptase polymerase chain reaction (RT‐PCR) and quantitative digital PCR for ALK fusion transcripts.^42^, ^96^, ^97^, ^100^, ^101^, ^102^, ^103^, ^104^, ^105^, ^106^ In ALCL, it has been shown that blood as a medium for minimal disease detection is more sensitive compared to bone marrow so that bone marrow punctures can be omitted for this purpose.^97^, ^101^, ^102^, ^105^ Determination of MDD, early MRD during front‐line therapy (before the second course of standard therapy) and during relapse therapy to follow the course of the disease can now be regarded as standard of care diagnostics and response evaluation improving clinical decision‐making.^103^, ^105^ For more details on MDD and MRD in ALCL, including discussion of techniques we refer to recent reviews and primary literature.^42^, ^54^, ^59^, ^62^, ^96^, ^97^, ^98^, ^99^, ^100^, ^101^, ^102^, ^104^, ^105^, ^106^ Technical issues regarding ALK‐antibody determination, interlaboratory or observer variations regarding real‐time quantitative PCR (RQ‐PCR) and histology, and the need for quality control and standardization led to selection of MDD and MRD as risk factors for patient selection or stratification in current clinical trials (ALCL‐VBL: *EudraCT*: 2017‐002935‐40; BrigaPED: NCT04925609).^107^ MRD could even serve as an end‐point in early clinical trials.

**TABLE 4:** Validated prognostic factors for risk stratification of ALK‐positive anaplastic large‐cell lymphoma treated with chemotherapy.

Risk factor		% patients with risk factor	EFS or PFS at 2–5 years (%)	Comment	Reference
Histological subtype	Lymphohistiocytic or small cell component (‘not common’)	35%	50%–55%	High interobserver variation	4
	Common	65%	80%–85%		
Qualitative MDD by RT‐PCR in blood at diagnosis	Positive	55%–60%	50%	International QC established	96, 97
	negative	40%–45%	80%–85%		
Quantitative MDD by RQ‐PCR in blood	≥10 NCN	25%–37%	32%–58%	High interlaboratory variation, no established QC	59, 62, 97, 98, 101
	<10 NCN	63%–75%	79%–89%		
Quantitative MDD by digital PCR in blood	e.g. ≥30 NCN	30%–35%	35%	International QC established	101, 102, 104
	<30 NCN	65%–70%	75%		
MRD before course 2 by qualitative PCR or digital PCR	Positive	20%–25%	20%	QC established Very high‐risk group	99, 100, 102
	Negative	75%	80%		
Anti‐ALK antibodies	<1/750	30%	40%	Assay includes subjective evaluation	36, 42, 43, 98
	>1/750	70%	80%		

Abbreviations: ALK, anaplastic lymphoma kinase; EFS, event‐free survival; MDD, minimal disseminated disease; MRD, minimal residual disease; PFS, progression‐free survival; QC, quality control; RQ‐PCR, real‐time quantitative PCR; RT‐PCR, reverse transcriptase polymerase chain reaction.

## CURRENT STANDARD THERAPY: FRONT‐LINE AND RELAPSE

### Initial treatment

Given that most chemotherapy regimens achieve comparable EFS rates, the optimal front‐line therapy for ALK‐positive ALCL can be considered the regimen that offers the shortest duration of therapy with the lowest cumulative doses of agents associated with a high risk of late effects. Currently, standard front‐line therapy consists of one of two regimens, depending on the availability of BV. In regions where BV is not available, the standard is six cycles of intensive chemotherapy without maintenance, as established by the ALCL99 trial. In regions where BV is available, the recommended approach is to incorporate BV into the ALCL99 backbone, following the results of the ANHL12P1.^59^

### Relapse treatment

Despite overall survival rates approaching 80% for relapsed patients, there is currently no standard of care for treatment. Based on the study from Knorr et al., relapses are thought of as low risk (>1 year from initial diagnosis), high risk (end of therapy but <1 year) and very high risk (on therapy). It should be noted that these risk groups are based on 5 months of treatment and cannot be extrapolated to more prolonged initial therapies or maintenance therapies.

### Initial therapy at relapse

The few children with a late relapse can be treated with 2 years of weekly vinblastine monotherapy.^49^ However, these data are from a study prior to the availability of BV and ALK inhibitors complicating the decision. Although there has not been a formal study, based on the response rates, low‐risk relapse patients might be adequately treated with either BV or an ALK inhibitor for a similar duration. For high‐risk and very high‐risk patients, the goal is to induce a remission. Based on its FDA approval for use in adults with relapsed ALCL, BV is frequently used in the United States in relapsed paediatric ALCL for patients who did not receive the agent during front‐line therapy. Although responses may be observed in patients who have received BV previously, its use may be less common due to possible cumulative toxicity and the availability of other active agents. For patients who have not received an ALK inhibitor, ALK inhibitors are often utilized. Crizotinib (US, EU) and alectinib (Japan) have indications for relapsed ALCL, but all of the ALK inhibitors have shown activity in this scenario. One advantage of brigatinib, alectinib or lorlatinib is that they penetrate the CNS, whereas crizotinib does not, but they come with their own side effect profiles. As noted earlier, there are multiple other options with less data and usually reserved for subsequent relapses.

#### Indication for consolidation by an allogenic HSCT for high‐risk and very high‐risk relapses

This is a question without an easy answer due to the lack of data. In light of the development of targeted drugs, the indication for consolidation of children with relapsed ALCL by an allogeneic HSCT with its risks is being questioned. There are reports of patients receiving ALK inhibitors and eventually stopping them with no recurrence of the disease, avoiding unnecessary toxicities (Table 3).^81^ There are also cases where the drug is stopped and the ALCL relapses immediately.^77^, ^88^ Unfortunately, it is not possible currently to determine the difference between the two groups. Therefore, other factors (e.g. availability of a donor, adverse effects of current treatment, pathological subtype, high vs. very high risk) create a setting for a nuanced conversation, as does the choice of conditioning in light of first experiences with reduced toxicity regimen.^46^, ^70^, ^71^ It should also be noted that currently an allogeneic HSCT is the only consolidation with a proven chance of cure for children with refractory ALCL.

Given the lack of a clear standard, treatment of patients with first relapse of an ALCL within a clinical trial is highly encouraged. Two trials with ALK inhibitors are open for recruitment: The post‐marketing trial CRISP with Crizotinib (EudraCT 2015‐005437‐53) and the phase I/II study BrigaPED testing brigatinib (NCT04925609). The trial NIVOALCL (NCT03703050) testing the efficacy of nivolumab has completed recruitment.

## CNS‐POSITIVE ALCL (INITIALLY OR AT RELAPSE)

Historically, central nervous system (CNS)‐positive ALCL was associated with a high risk of failure and death both initially and at relapse.^69^, ^108^, ^109^ Intensive, CNS‐directed chemotherapy was used for those patients from the 1990s.^24^, ^56^ In more than half of published patients, craniospinal irradiation was added^19^, ^21^, ^23^, ^24^, ^25^, ^52^, ^109^ although the role of craniospinal radiotherapy for the therapy of CNS‐positive ALCL remains unclear.^56^, ^109^ This approach led to an EFS of approximately 50% in newly diagnosed patients.^109^ Vinblastine and the first targeted drugs, BV and crizotinib, do not penetrate the CNS adequately so CNS progressions were observed even in CNS‐negative relapsed disease.^110^ The use of CNS‐penetrable second‐ or third‐generation ALK‐inhibitors in children with CNS relapse led to remission and cure in nine of 10 children with CNS‐relapse in a case series.^111^ In recent years with ALK inhibitors' availability, retrospective analyses suggest that the outcome of patients with CNS‐positive relapse improved.^82^, ^112^ Given the exceeding rarity of CNS‐positive ALCL, the current poor outcome, the risk for acute toxicity and the late effects of the front‐line therapy, there is an urgent need to test CNS‐penetrable ALK inhibitors in front‐line therapy of CNS‐positive ALCL patients.

## CURRENT CLINICAL TRIALS WITH STRATIFICATION CRITERIA

The only open international trial for initial treatment is ALCL‐VBL which is investigating whether children with a low risk of relapse (patients with stage I–III disease who are MDD negative in blood at diagnosis) can be cured with 24 months of weekly vinblastine monotherapy (*EudraCT*: 2017‐002935‐40).

For children and adolescents with R/R ALCL, two trials with ALK inhibitors are currently open for recruitment: the post‐marketing trial CRISP with Crizotinib (EudraCT 2015‐005437‐53) and the phase 1/2 study BrigaPED testing brigatinib (NCT04925609). The trial NIVO‐ALCL (NCT03703050) testing the efficacy of Nivolumab has completed recruitment.

MDD and MRD are utilized for risk stratification of newly diagnosed patients for clinical trials. In some areas of the world (e.g. most of Europe), clinical decisions are already based on MDD and MRD outside of a clinical study. In the United States, these parameters are not currently used in routine clinical practice, but this is expected to change given the results of the ANHL12P1 trial, which demonstrated distinct risk categories by utilizing MDD.

## CURRENT CLINICAL NEED AND OUTLOOK

ALCL is a unique cancer in that current successful relapse therapy is often less toxic than the initial therapy. Recent retrospective analyses suggest that patients with ALCL are likely to achieve cure even after a second or subsequent relapse.^49^, ^82^, ^112^ Resistance in ALCL appears to be an innate biological characteristic rather than induced by therapy, offering the opportunity for successful retreatment. Taken together, it seems necessary and possible to test a less toxic backbone and eventually ‘chemotherapy‐free’ modalities. Patients with refractory/resistant disease in need of new approaches can now be identified using MDD and MRD. Targeted drugs, such as ALK inhibitors, have shown significant efficacy in ALCL. However, their role in front‐line therapy remains unclear, and no ongoing front‐line trial incorporating ALK inhibitors is available in Europe or the United States, despite evidence of their safety and efficacy.

Based on the available data and options, we could envision a treatment strategy of induction of clinical remission with either BV or a CNS‐penetrable ALK inhibitor; a deep remission could be established by several months or even a few years of ALK inhibitor therapy. Consolidation could then be individualized based on the patients' immune response against ALK as measured by either the ‘control’ of disease by MRD or the existence of ALK‐specific T cells and may include vaccination with ALK epitopes (either mRNA or peptides) or a low‐toxicity allogeneic HSCT based on the individual immune response against the lymphoma.

Importantly, the design of clinical trials testing novel approaches must account for the fact that ALK‐positive ALCL is an orphan disease with less than 200 children and adolescents diagnosed annually across Europe and the United States. To facilitate a reasonable timeframe for trial completion, innovative trials are essential. Furthermore, despite ALK‐positive ALCL being the prototypical cancer dependent on ALK signalling, the interest of pharmaceutical companies to develop drugs towards licensing in orphan diseases seems to be limited. Collaborative efforts are critical to the successful development of new drugs and strategies for ALCL.

## QUESTIONS FOR FUTURE DEVELOPMENT OF THERAPY

Collectively, the current state of ALCL treatment and the availability of effective agents underscore the urgent need for clinical trials to evaluate novel, less toxic and more effective therapeutic approaches. Key questions include:

- Can newly diagnosed patients who are MDD negative be cured with minimal or no chemotherapy (e.g. BV) in combination with an ALK inhibitor or with ALK inhibitors as single drugs given for a defined time?
- Can the EFS for newly diagnosed patients who are MDD positive be improved by adding an ALK inhibitor?
- Are high‐risk relapses best managed with an HSCT?
  ○ Is reduced conditioning as effective as total body irradiation?
- Can using an ALK inhibitor for a specific time avoid the need for a HSCT in relapsed ALCL?
  ○ If this strategy is employed and the patient relapses a second time, does a HSCT have the same efficacy?
- Does the use of an ALK inhibitor for years before an allogeneic HSCT decrease or increase survival versus up‐front HSCT?
- Can ALK inhibitors be safely and effectively used as maintenance therapy following HSCT?

Given our current knowledge, these questions are feasible to address and essential for advancing treatment outcomes for patients with ALCL.

Despite its status as an orphan disease, significant advancements in the treatment of children and adolescents with ALCL have been achieved over the past few decades. Paediatric oncologists have demonstrated that clinical trials using targeted agents in rare cancers are not only possible but also lead to transformative progress. With sustained collaboration among cooperative trial groups and support from the pharmaceutical industry and regulators, the pivotal questions can be answered, leading to a new era in the treatment of ALCL.

## AUTHOR CONTRIBUTIONS

WW and EJL structured and wrote the review.

## CONFLICT OF INTEREST STATEMENT

The authors declare no conflict of interest.
